# The serum uric acid to apolipoprotein A1 ratio is independently correlated with metabolic dysfunction-associated steatotic liver disease in type 2 diabetes mellitus: findings from a single national metabolic management center cohort

**DOI:** 10.3389/fendo.2025.1619003

**Published:** 2025-06-04

**Authors:** Xiu Li Guo, Mei Tu, Xiu Ping Qiu, Wei Wang

**Affiliations:** National Metabolic Management Center, Longyan First Affiliated Hospital of Fujian Medical University, Longyan, Fujian, China

**Keywords:** serum uric acid to apolipoprotein A1 ratio, metabolic dysfunction-associated steatotic liver disease, insulin resistance, type 2 diabetes mellitus, apolipoprotein A1

## Abstract

**Background:**

Recent evidence suggests that the serum uric acid to apolipoprotein A1 ratio (UAR) may be a novel biomarker for metabolic dysfunction-associated steatotic liver disease (MASLD). This study aims to investigate the relationship between UAR and MASLD, and compare the diagnostic ability of UAR with other insulin resistance-related markers for MASLD in individuals with type 2 diabetes mellitus (T2DM).

**Method:**

A cohort of 1,019 individuals with T2DM was recruited from the National Metabolic Management Center of our hospital. Unenhanced abdominal CT scans were performed to evaluate liver steatosis for diagnosing MASLD. The association between UAR and the risk of MASLD was analyzed using weighted binomial logistic regression, restricted cubic splines (RCS), and subgroup analysis. Receiver operating characteristic (ROC) curve analysis was conducted to compare the diagnostic performance of UAR with other insulin resistance-related markers, including the serum uric acid to high-density lipoprotein cholesterol ratio (UHR), triglyceride to high-density lipoprotein cholesterol ratio (THR), and triglyceride to apolipoprotein A1 ratio (TAR).

**Results:**

Participants in the MASLD group exhibited elevated UAR levels. After full adjustments for potential confounders, UAR remained independently associated with MASLD (*OR:* 1.65, 95% CI: 1.45-1.89, *P* < 0.001). Subgroup analyses revealed that this association was consistent across various subgroups, including sex, drinking status, hypertension, lipid-lowering therapy, and body mass index (*P* < 0.05). RCS analysis demonstrated a linear increase in the risk of MASLD with higher UAR levels (*P* for nonlinear = 0.319). ROC curve analysis indicated that UAR provided good diagnostic performance for MASLD (AUC:0.777, 95% CI: 0.749- 0.805), comparable to TAR (AUC difference: -0.003, 95% CI: -0.033-0.026, *P* = 0.818) and superior to UHR (AUC difference: 0.043, 95% CI: 0.019-0.067, *P* < 0.001) and THR (AUC difference: 0.035, 95% CI: 0.019-0.067, *P* = 0.047).

**Conclusion:**

UAR was independently associated with MASLD and demonstrated significant diagnostic value, indicating that UAR could be a cost-effective biomarker to help identify high-risk individuals for MASLD.

## Introduction

Metabolic dysfunction-associated steatotic liver disease (MASLD), previously referred to as non-alcoholic fatty liver disease (NAFLD), is characterized by steatotic liver disease accompanied by one or more cardiometabolic risk factors without harmful alcohol consumption. Type 2 diabetes mellitus (T2DM) is a significant metabolic disease that influences the natural progression of MASLD. It can accelerate the progress of MASLD into metabolic dysfunction-associated steatohepatitis (MASH), fibrosis, cirrhosis, or even severe MASH-related hepatocellular carcinoma (1). Consequently, the global prevalence of MASLD and MASH in individuals with T2DM can be as high as 65.04% and 35.54%, respectively (2). Furthermore, the impact of MASLD on T2DM is profound. Beyond causing liver damage, MASLD can significantly increase insulin resistance, worsen blood glucose control, and even elevate the cardiovascular morbidity (3) and all-cause mortality (4). Due to the high prevalence and significant impact of T2DM, there is an urgent need for early screening of individuals at high risk for MASLD. Blood biomarkers resulting from the pathophysiological changes associated with MASLD may serve as a reliable method to identify high-risk individuals. Insulin resistance disrupts lipid and hepatic glucose metabolism, promoting lipogenesis, a key pathogenic factor in the progression of MASLD. Several clinical studies have identified various insulin resistance markers, such as the serum uric acid to high-density lipoprotein cholesterol ratio (UHR), triglyceride to high-density lipoprotein cholesterol ratio (THR), and triglyceride-glucose (TyG) index based on lipid profiles, blood glucose levels, and uric acid concentrations, to help identify high-risk groups for MASLD (5–7).

Apolipoprotein A1 (APOA1) is the primary apolipoprotein of plasma high-density lipoprotein (HDL-c), renowned for its well-established cardioprotective functions. Beyond its role in HDL-c, APOA1 plays a multifaceted role in anti-inflammation, anti-insulin resistance, anti-atherosclerosis, and other processes (8, 9). The role of peroxisome proliferator-activated receptors (PPARs) in the pathogenesis of NAFLD, particularly through insulin resistance, has been increasingly recognized (10). Chen et al. demonstrated that APOA1 functions as a key hub protein linking PPARs to NAFLD by beneficially modulating 16 out of 21 NAFLD upstream regulators. Elevated levels of APOA1 were found to be inversely associated with the prevalence of NAFLD, suggesting that increased APOA1 levels may offer a protective effect against the disease (11). Our previous studies have shown that APOA1 is a more effective component of MASLD biomarkers than HDL-c. Specifically, the monocyte-to-APOA1 ratio (MAR) and the triglyceride-to-APOA1 ratio (TAR) demonstrated superior diagnostic accuracy compared to the monocyte-to-HDL-c ratio (MHR) and THR (12, 13). The UHR, as a new marker of insulin resistance, has been demonstrated to be associated with various cardiometabolic diseases like metabolic syndrome (14) and MASLD (5). However, it remains unclear whether the serum uric acid-to-APOA1 ratio (UAR) offers superior diagnostic capability compared to UHR in identifying MASLD. Hence, this study aims to investigate the relationship between UAR and MASLD and compare the diagnostic ability of UAR with UHR, THR, and TAR for MASLD in T2DM.

## Materials and methods

### Study cohort enrollment

This cross-sectional study was conducted at the National Metabolic Management Center (MMC) of Longyan First Affiliated Hospital of Fujian Medical University. Participants with T2DM who expressed willingness to join the MMC management program were prospectively recruited for this study between June 2022 and December 2024. The ethics committee of our hospital approved this program in strict adherence to the principles outlined in the Declaration of Helsinki (IC-2022-009). All participants were fully informed about the study objectives and provided written consent. Participants were excluded from the final analysis based on these criteria: 1) Heavy alcohol consumption (defined as daily alcohol intake of ≥30 g for men and ≥20 g for women). 2) Presence of liver comorbidities, including liver malignancy, autoimmune hepatitis, viral hepatitis, or individuals receiving treatments (e.g., estrogens, tamoxifen, antiretroviral drugs, methotrexate, antipsychotics, or glucocorticoids) that could contribute to steatotic liver disease. 3) Receiving UA-lowering therapy (e.g., allopurinol, febuxostat, or benziodone). 4) Accompanied by acute illnesses that can lead to dramatic changes in UA and lipids (e.g., diabetic ketoacidosis, hyperglycemic hyperosmolar syndrome, and hyperlipidemic pancreatitis). 5) Pregnant women or those with incomplete data. After applying the exclusion criteria, 122 participants were excluded, and 1019 participants were included in the final analysis (**Supplementary Figure 1**).

### Data collection

Demographic data, including sex, age, duration of diabetes, medication use, alcohol consumption, and medical history, were collected by the researchers using standardized questionnaires. Additionally, anthropometric measurements, including weight, height, waist circumference (WC), and blood pressure (BP), were recorded by trained nurses. Body mass index (BMI) was calculated using the formula: weight (kg) divided by height squared (m²). Following enrollment, fasting blood samples were collected from all participants after a minimum 8-hour fasting period and sent to the central laboratory for analysis. Laboratory assessments included measurements of UA, creatinine, alanine aminotransferase, aspartate aminotransferase (AST), albumin, TG, total cholesterol (TC), HDL-c, low-density lipoprotein cholesterol (LDL-c), APOA1, fasting blood glucose, and HbA1c. Biochemical parameters were assessed using an automated biochemical analyzer (Roche Diagnostics Corporation), while HbA1c levels were measured via high-performance liquid chromatography with a D10 set (Bio-Rad). All collected data were stored in the Specific MMC management system for subsequent analysis.

### Insulin resistance-related index assessment

UAR, UHR, TAR, and THR were separately calculated using the following formulas: 1) UAR (%) = serum UA (mg/dl)/APOA1 (mg/dl) x 100; 2) UHR (%) = serum UA (mg/dl)/HDL-c (mg/dl) x 100; 3) TAR= serum TG (mg/dl)/APOA1 (mg/dl); 4) THR= serum TG (mg/dl)/HDL-c (mg/dl).

### Assessment of MASLD

The assessment process of MASLD was based on the clinical practice guidelines for managing MASLD, developed by the EASL-EASD-EASO (15). Individuals with T2DM who exhibit liver steatosis, as detected through imaging or liver pathology, and who do not have harmful alcohol intake or other identifiable causes of liver steatosis, are identified as having MASLD. As the MMC program necessitates the enhancement of abdominal CT to assess pancreatic morphology, all participants were evaluated for liver steatosis using non-enhanced CT imaging. An experienced radiologist independently assessed the presence of liver steatosis using CT hepato-spleen attenuation measurements (CT_L-S_) using a standardized method without knowledge of the clinical data. Liver attenuation was measured by calculating the average HU value from three 3 cm² circular regions of interest (ROIs). These ROIs were manually placed in the left hepatic lobe, the anterior segment of the right hepatic lobe, and the posterior part of the right hepatic lobe. Similarly, splenic attenuation was determined by averaging the HU values from three 2 cm² ROIs located in the upper, middle, and lower thirds of the spleen. The CT_L-S_ is the ratio of the liver’s average attenuation value to the spleen’s average attenuation value. CT_L-S_ values less than 1.0 were identified as liver steatosis.

### Statistical analysis

Statistical analyses were performed using SPSS version 29.0 or R language version 4.2.3 software. Independent samples T-tests or chi-squared (χ²) tests were separately conducted to compare the differences in baseline characteristics between the MASLD and non-MASLD groups. 18 variables, including gender, age, diabetic duration, drinking (yes or no), hypertension (yes or no), lipid-lowering therapy (yes or no), receiving TZDs, SGL-T2, or GLP1-RAs (yes or no), WC, BMI, AST, ALT, creatinine, albumin, HbA1c, TC, HDL-c, LDL-c, and TG, were included in Lasso regression to identify the most relevant covariates associated with MASLD and address collinearity issues. Weighted binomial logistic regression and restricted cubic splines (RCS) analysis were used to evaluate the association between UAR and the risk of MASLD, adjusting for the variables retained in the final Lasso model. Additionally, model fitness was assessed by the Hosmer-Lemeshow test. A receiver operating characteristic (ROC) curve analysis was conducted to compare the diagnostic performance of UAR, UHR, TAR, and THR for MASLD. The *P* < 0.05 was considered statistically significant.

## Result

### Clinical characteristics of study cohort

**Figure 1:**
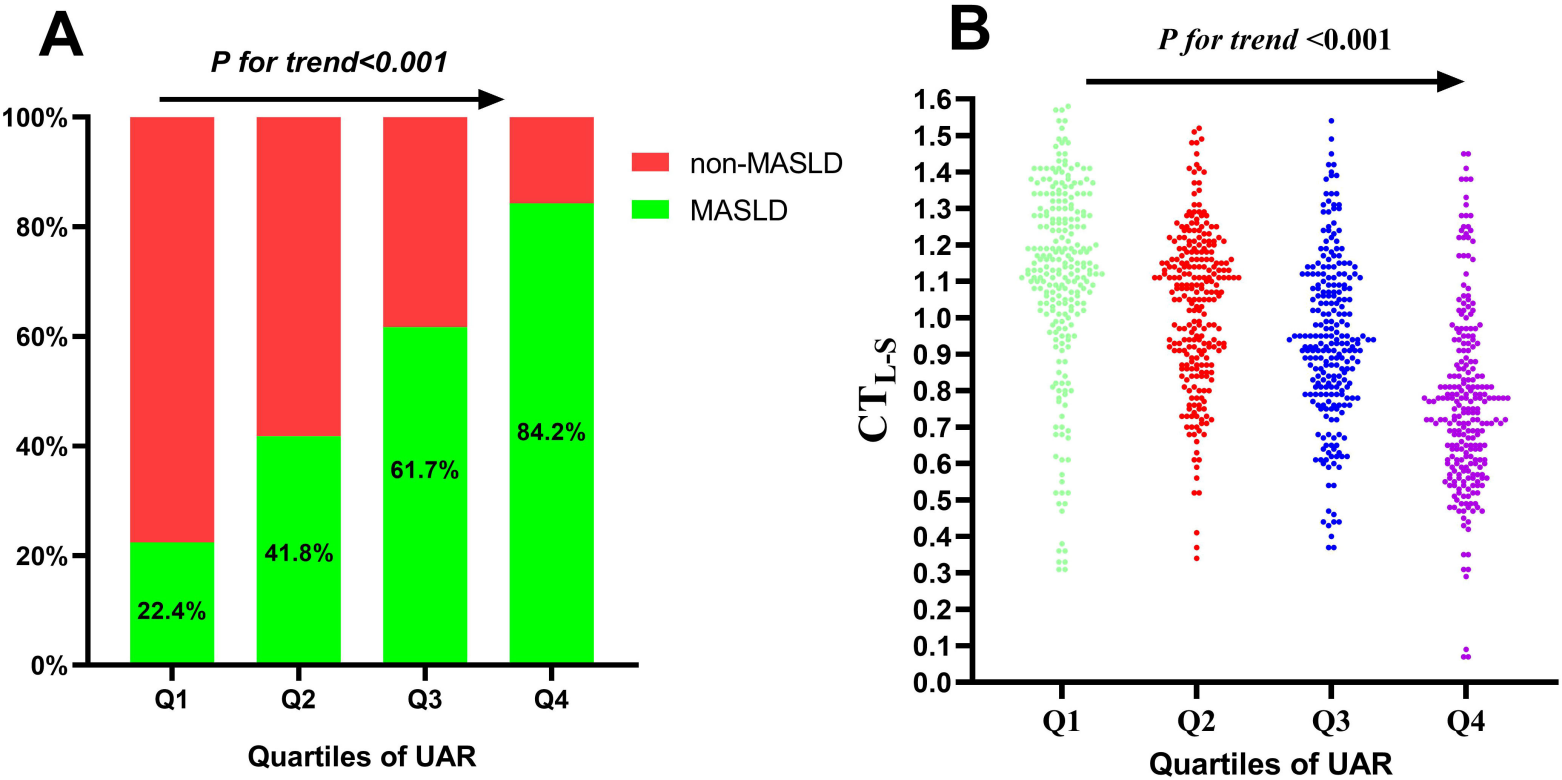
Prevalence of MASLD **(A)** and distribution of CTL-S **(B)** based on quartiles of UAR. MASLD, metabolic dysfunction-associated steatotic liver disease; CTL-S, CT hepato-spleen attenuation measurements; UAR, serum uric acid to apolipoprotein A1 ratio.

The study cohort comprised 533 individuals (52.6%) diagnosed with MASLD. The mean age of participants was 56.0 ± 8.2 years, and the mean duration of diabetes was 6.4 ± 3.5 years. **Table 1** summarizes the comparison of clinical characteristics between the MASLD and non-MASLD groups. Participants in the MASLD cohort exhibited a significantly longer diabetic duration and elevated BMI, WC, TG, LDL-c, UA, AST, ALT, and insulin resistance-related indexes like UAR, UHR, TAR, and THR (*P* < 0.05). Conversely, participants in the MASLD group demonstrated reduced levels of HDL-c and APOA1(*P* < 0.05). Additionally, participants in higher quartiles of UAR had an increased prevalence of MASLD (**Figure 1A**) and decreased levels of CT_LS_ (**Figure 1B**).

**Table 1:** Comparison of baseline characteristics between MASLD and non-MASLD groups.

Characteristics	Total (n=1019)	MASLD (n=533)	Non-MASLD (n=486)	*P* value
Age (year)	56.0 ± 8.2	55.9 ± 8.0	56.1 ± 8.3	0.914
Diabetic duration (year)	6.4 ± 3.5	6.9 ± 3.3	5.9 ± 3.6	<0.001
BMI (kg/m^2^)	24.5 ± 4.9	25.5 ± 3.0	23.2 ± 2.5	<0.001
WC (cm)	85.9 ± 6.9	88.7 ± 7.0	82.9 ± 5.4	<0.001
HbA1c (%)	8.7 ± 2.0	8.7 ± 2.0	8.7 ± 1.9	0.919
TG (mg/dl)	84.1 ± 53.5	101.5 ± 57.8	64.9 ± 43.4	<0.001
TC (mg/dl)	202.7 ± 45.2	200.8 ± 45.1	204.9 ± 45.4	0.141
HDL-c (mg/dl)	42.3 ± 9.2	38.9 ± 7.9	46.11 ± 7.9	<0.001
LDL-c (mg/dl)	134.0 ± 36.3	136.2 ± 36.2	131.7 ± 36.3	0.048
APOA1 (mg/dl)	126.6 ± 26.3	115.3 ± 21.1	139.1 ± 25.7	<0.001
UA (mg/dl)	5.84 ± 1.44	6.26 ± 1.50	5.38 ± 1.22	<0.001
Creatinine (umol/L)	70.0 ± 13.2	69.4 ± 13.3	70.7 ± 13.0	0.093
ALT (U/L)	39.9 ± 9.0	41.6 ± 9.4	37.9 ± 8.1	<0.001
AST (U/L)	37.4 ± 8.9	39.1 ± 8.4	35.3 ± 7.8	<0.001
Albumin (g/L)	40.0 ± 2.3	39.9 ± 2.4	40.1 ± 2.2	0.483
IR-related indexes				
UAR (%)	4.91 ± 2.38	5.72 ± 2.84	4.01 ± 1.24	<0.001
UHR (%)	166.5 ± 37.6	16.91 ± 5.69	12.37 ± 4.48	<0.001
TAR	12.1 ± 4.6	0.95 ± 0.84	0.49 ± 0.34	<0.001
THR	0.22 ± 0.34	2.85 ± 1.98	1.64 ± 1.57	<0.001
Gender, n (%)				
Men	509 (49.9)	272 (51.0)	237 (48.8)	0.470
Women	510 (50.1)	261 (49.0)	249 (51.2)	
Hypertension, n (%)				
With	351 (34.4)	247 (46.3)	104 (21.4)	<0.001
Without	668 (65.6)	286 (53.7)	382 (78.6)	
Drinking, n (%)				
With	357 (35.0)	237 (44.5)	120 (24.7)	<0.001
Without	662 (65.0)	296 (55.5)	366 (75.3)	
Receiving lipid-lowing therapy, n (%)				
With	314 (30.8)	136 (25.6)	178 (36.6)	<0.001
Without	705 (69.2)	397 (74.4)	308 (63.4)	
Receiving TZDs, SGL-T2, or GLP1-RAs, n (%)				
With	278 (27.3)	148 (27.8)	130 (26.7)	0.715
Without	741 (72.7)	385 (72.2)	356 (73.3)	

Data are expressed as n (%) or mean ± standard deviation. BMI, body mass index; WC, waist circumference; HbA1c, glycated hemoglobin; UA, uric acid; TG, triglyceride; TC, total cholesterol; HDL-c, high-density lipoprotein cholesterol; LDL-c, low-density lipoprotein cholesterol; APOA1, apolipoprotein A1; ALT, alanine aminotransferase; AST, aspartate aminotransferase; UAR, serum uric acid to apolipoprotein A1 ratio; UHR, serum uric acid to high-density lipoprotein cholesterol ratio; TAR, triglyceride to apolipoprotein A1 ratio; THR, triglyceride to high-density lipoprotein cholesterol ratio; MASLD, metabolic dysfunction-associated steatotic liver disease; TZDs, Thiazolidinediones; SGL-T2, sodium-dependent glucose transporters 2 inhibitors; GLP1-RAs, Glucagon-like peptide-1 receptor agonists.

### Screening of confounding variables using Lasso regression

**Figure 2:**
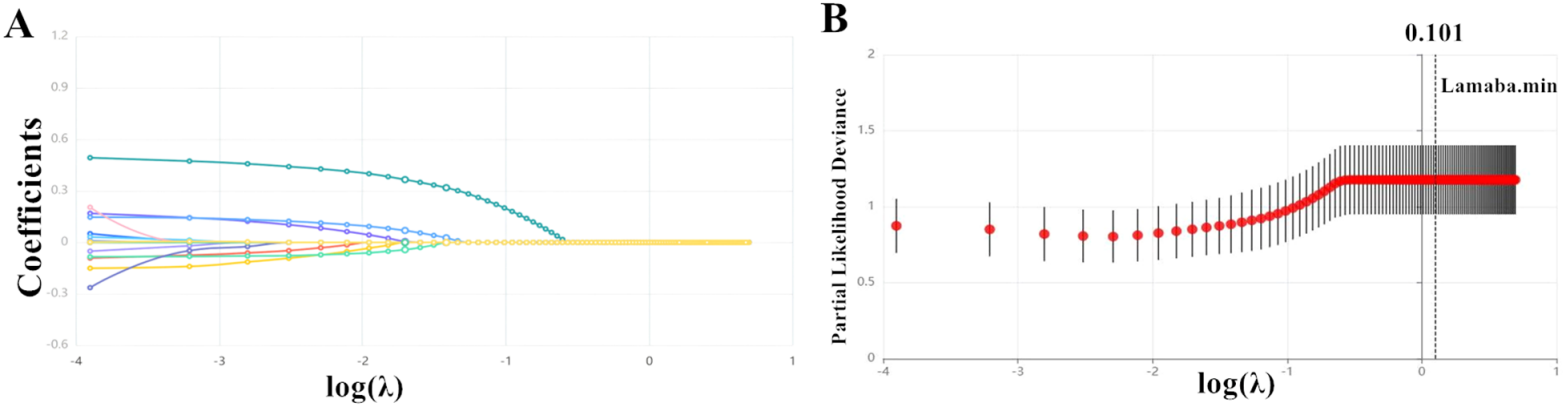
The variation in the variable coefficients **(A)** and the process of selecting the optimal value for the parameter λ in the Lasso regression model using the cross-validation method **(B)**.

Lasso regression analysis was conducted to identify the most relevant covariates associated with MASLD. The variation characteristics of the coefficients for the selected variables were depicted in **Figure 2A**. As illustrated in **Figure 2B**, the optimal model, which achieved the best performance with the fewest variables, was obtained at a λ value of 0.101 (Log λ = -2.293). The variables that remained in the final model included age, TG, TC, HDL-c, LDL-c, WC, creatinine, AST, and ALT.

### Association between UAR and the risk of MASLD

**Figure 3:**
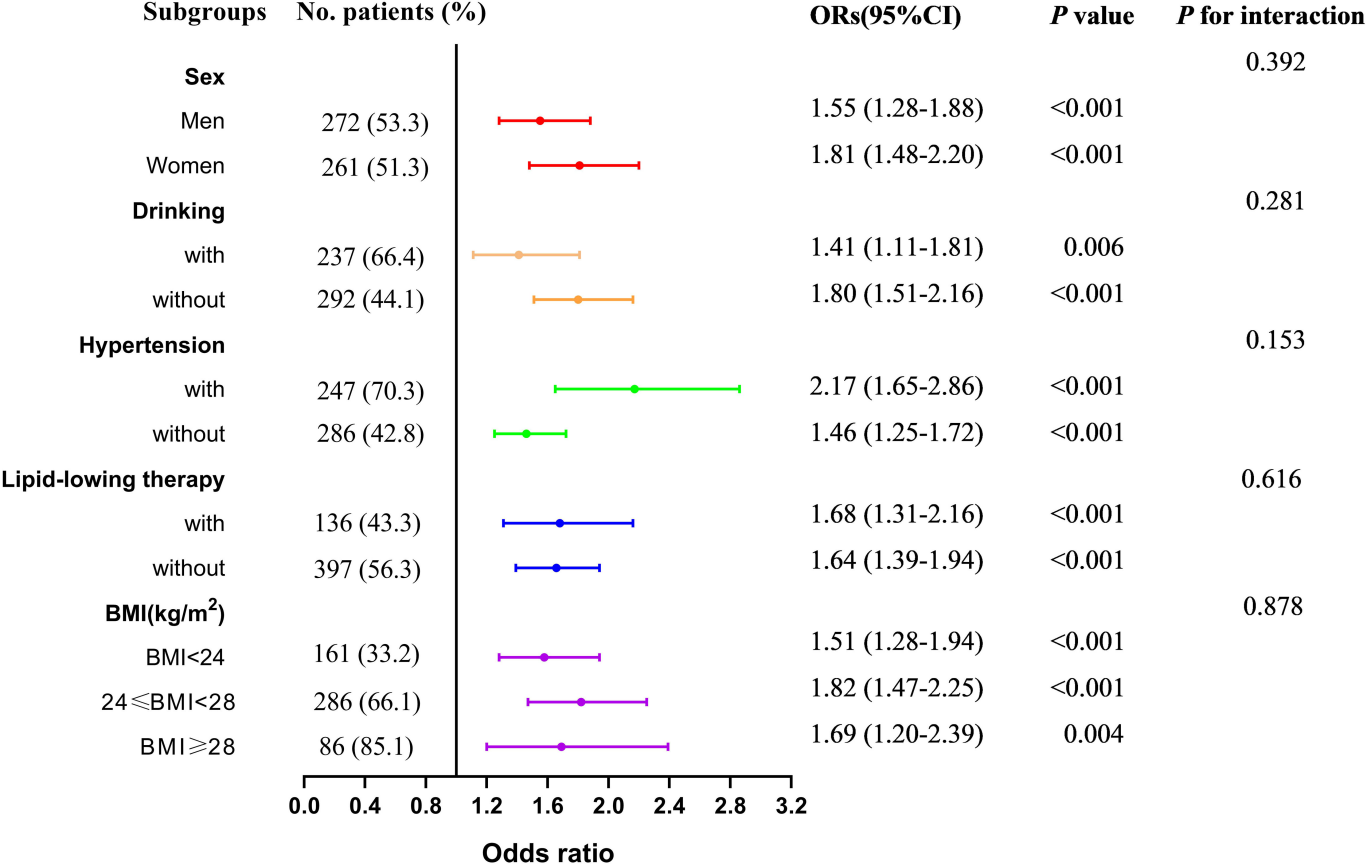
Subgroup analysis for the association between UAR and the risk of MASLD across sex, drinking, hypertension, lipid-lowering therapy, and BMI. Notes: Model adjusted age, waist circumference, triglyceride, total cholesterol, low-density lipoprotein cholesterol, high-density lipoprotein cholesterol, creatinine, alanine aminotransferase, and aspartate aminotransferase. MASLD, metabolic dysfunction-associated steatotic liver disease; UAR, serum uric acid to apolipoprotein A1 ratio. BMI, body mass index.

**Figure 4:**
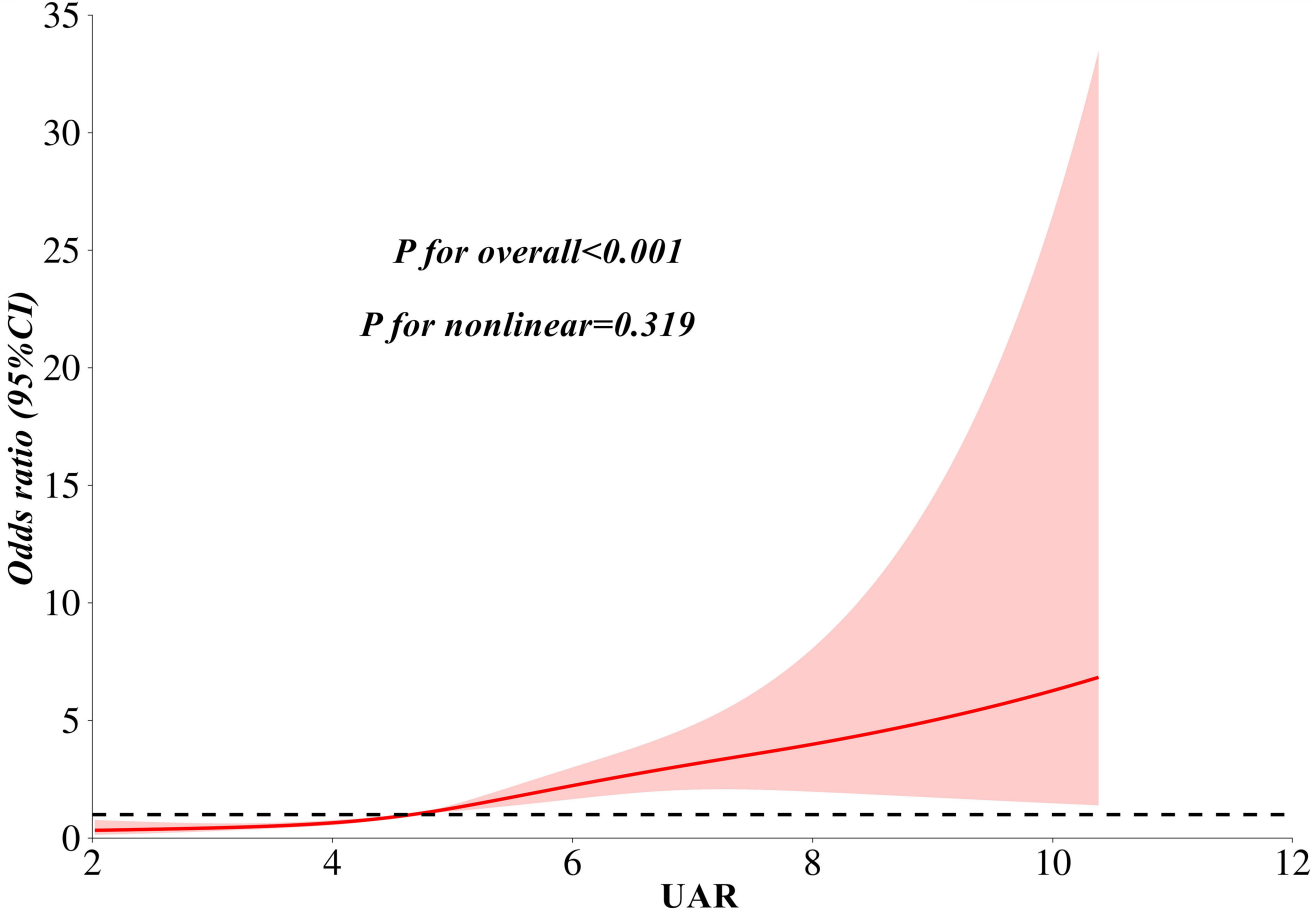
A linear association between UAR and the risk of MASLD as analyzed by restricted cubic splines analysis. Notes: Model adjusted for age, waist circumference, triglyceride, total cholesterol, low-density lipoprotein cholesterol, high-density lipoprotein cholesterol, creatinine, alanine aminotransferase, and aspartate aminotransferase. MASLD, metabolic dysfunction-associated steatotic liver disease; UAR, serum uric acid to apolipoprotein A1 ratio.

**Table 2** presents the association between UAR and the risk of MASLD, analyzed using weighted binomial logistic regression across three different models. Based on the final model of Lasso Regression, study covariates were incorporated into three models for the association between UAR and MASLD. Crude was unadjusted. Model 1^a^ was adjusted for age, TG, TC, HDL-c, LDL-c, WC, and creatinine. Model 2^b^ additionally adjusted for AST and ALT. After full adjustments in Model 2^b^, participants in the third and fourth quartiles of UAR had a significantly increased risk of MASLD compared to those in the first quartile (*P* < 0.001). Furthermore, UAR contributed to an independent risk factor for MASLD (*OR:* 1.65, 95% CI: 1.45-1.89, *P* < 0.001). Each SD increase in UAR was associated with a 232% higher risk of MASLD (*OR:* 3.32, 95% CI: 2.41-4.57, *P* < 0.001). Moreover, a significant dose-response relationship was also observed (*P*<0.001). **Figure 3** presents the results of subgroup analysis for the association between UAR and the risk of MASLD. The findings indicated that increased UAR remained significant with MASLD across the subgroup of sex, drinking, hypertension, lipid-lowering therapy, and BMI (all *P* < 0.05 and *P* for interaction >0.05). Furthermore, the RCS analysis also observed a linear increasing association between UAR and the risk of MASLD (**Figure 4**).

**Table 2:** Weighted binomial logistic regression analysis for the correlation of UAR with MASLD.

Variable	Crude		Model 1^a^		Model 2^b^	
	OR (95%CI)	*P* value	OR (95%CI)	*P* value	OR (95%CI)	*P* value
Per SD increase	6.45 (4.89-8.53)	<0.001	3.65 (2.67-4.98)	<0.001	3.32 (2.41-4.57)	<0.001
Overall	2.19 (1.95-2.46)	<0.001	1.72 (1.51-1.96)	<0.001	1.65 (1.45-1.89)	<0.001
Q1	Ref. (1.0)		Ref. (1.0)		Ref. (1.0)	
Q2	1.70 (1.13-2.56)	0.011	1.49 (0.96-2.31)	0.072	1.48 (0.95-2.32)	0.086
Q3	3.40 (2.24-5.16)	<0.001	2.95 (1.89-4.62)	<0.001	2.82 (1.78-4.47)	<0.001
Q4	9.60 (5.94-15.52)	<0.001	6.96 (4.19-11.57)	<0.001	6.20 (3.69-10.42)	<0.001
*P* for trend	<0.001		<0.001		0.001	

Crude: unadjusted. Model 1^a^: adjusted for age, waist circumference, triglyceride, total cholesterol, low-density lipoprotein cholesterol, high-density lipoprotein cholesterol, and creatinine. Model 2^b^: additionally adjusted for alanine aminotransferase and aspartate aminotransferase.

UAR, serum uric acid to apolipoprotein A1 ratio; MASLD, metabolic dysfunction-associated steatotic liver disease.

### Diagnostic ability of UAR for MASLD

**Figure 5:**
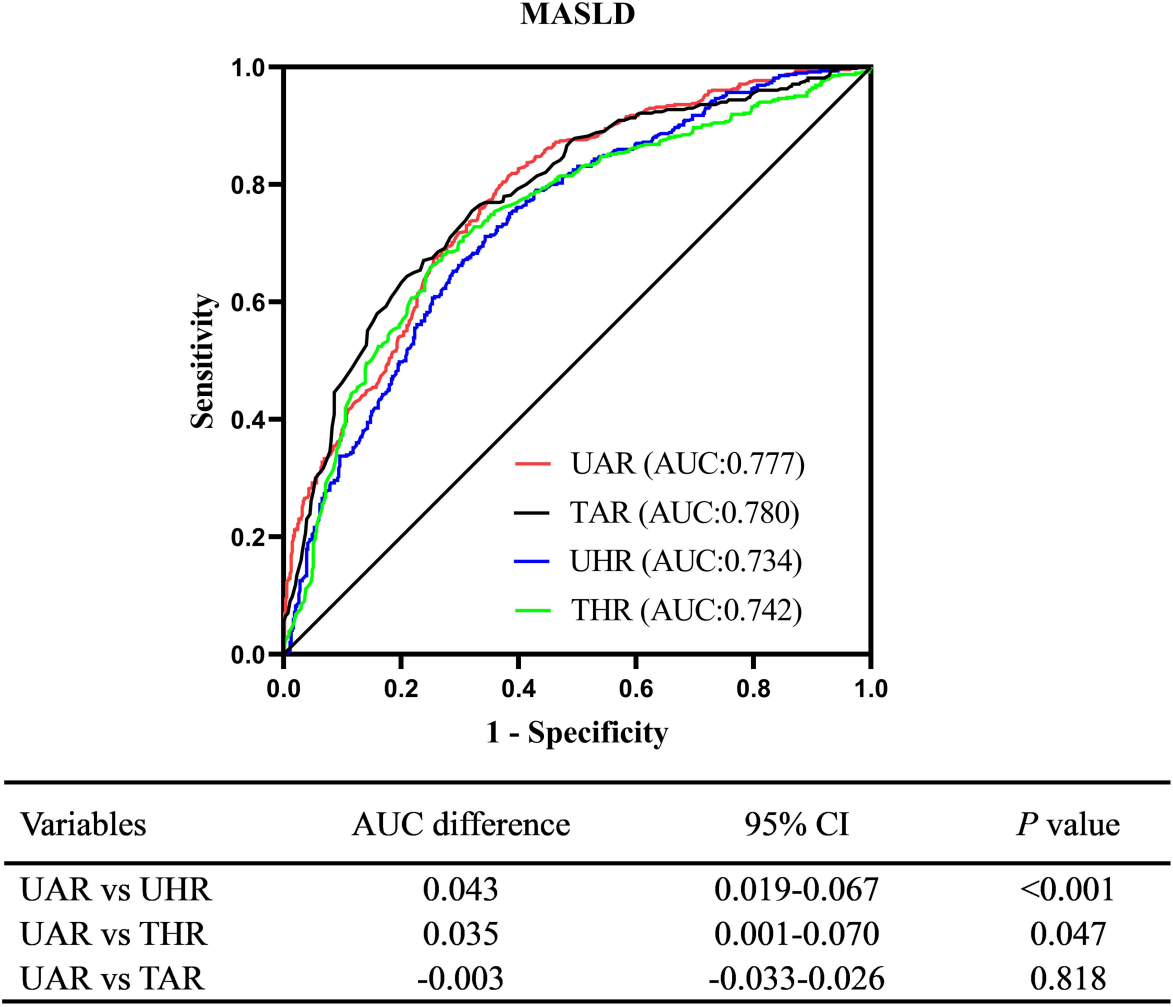
Comparison of diagnostic ability for MASLD between UAR, UHR, THR, and TAR. UAR, serum uric acid to apolipoprotein A1 ratio; UHR, serum uric acid to high-density lipoprotein cholesterol ratio; TAR, triglyceride to apolipoprotein A1 ratio; THR, triglyceride to high-density lipoprotein cholesterol ratio.

ROC curve analysis revealed a good diagnostic value of UAR for MASLD. The AUC (95% CI) of UAR identifying MASLD was 0.777 (0.749-0.805). The optimal cut-off value of UAR was 4.94%, with a sensitivity of 62.9% and a specificity of 80.5%. **Figure 5** illustrates the comparison of diagnostic ability for MASLD between UAR, UHR, THR, and TAR using DeLong analysis. The results indicated that UAR demonstrated similar diagnostic performance to TAR (AUC difference: -0.003, 95% CI: -0.033-0.026, *P* = 0.818). However, UAR was superior to UHR (AUC difference: 0.043, 95% CI: 0.019-0.067, *P* < 0.001) and THR (AUC difference: 0.035, 95% CI: 0.019-0.067, *P* = 0.047).

## Discussion

MASLD is a novel classification of steatotic liver disease associated with metabolic syndrome, which exerts significant effects on T2DM with an increased risk of extrahepatic outcomes. Simple and non-invasive screening of high-risk populations is crucial for the early diagnosis and effective management of MASLD. This study investigates the association between UAR and MASLD, comparing the diagnostic performance of UAR with UHR, THR, and TAR in T2DM. The results showed that participants in the MASLD cohort had a high level of UAR. Moreover, UAR was independently correlated with the risk of MASLD, as evidenced by weighted binomial logistic regression, RCS analysis, and subgroup analysis across sex, drinking habits, hypertension, lipid-lowering therapy, and BMI. Furthermore, UAR exhibited strong diagnostic performance for MASLD, comparable to TAR and superior to UHR and THR.

The availability of improved treatment methods highlights the importance of early identification of high-risk individuals for MASLD, as treatments such as lifestyle interventions, weight loss, pioglitazone, sodium-dependent glucose transporters 2 inhibitors, and glucagon-like peptide-1 receptor agonists can positively influence the disease process and are expected to prevent related clinical outcomes (16–19). While liver biopsy remains the gold standard for diagnosing and staging liver fibrosis severity, its invasive nature makes it unacceptable to the general population and inconvenient for use in primary care. Although guidelines recommend imaging as a detection method for steatotic liver, its high cost and potential radiation exposure may limit its use in large-scale screening of the general population. Various societies recommend non-invasive biomarkers based on combinations of blood tests for the early diagnosis of MASLD, which are generally well-accepted by the general population and can be performed repeatedly at an affordable cost. Metabolic perturbations, lipotoxicity, insulin resistance, and systemic inflammation lie the foundation for the development of MASLD (20, 21). Consequently, several inflammatory and insulin resistance-related markers for MASLD, based on metabolic profiles and blood cell combinations, have been investigated in previous studies. Wang et al. observed that specific systemic immune biomarkers, such as the systemic immune-inflammation index (SII), neutrophil-to-lymphocyte ratio (NLR), and neutrophil-to-albumin ratio (NPAR), were significantly associated with an increased risk of MASLD and demonstrated prognostic utility for the condition (22). In a subsequent study, Dong et al. further highlighted the predictive value of NPAR, NLR, and SII for all-cause mortality and cardiovascular disease in patients with MASLD (23). Similarly, Zhu et al. and Lu et al. identified the neutrophil-to-high-density lipoprotein cholesterol ratio (NHR) as a potential biomarker for MASLD, noting that reducing NHR levels may help decrease the incidence of MASLD and related complications (24, 25). Additionally, multiple clinical studies have established an independent association between the MHR and the risk of NAFLD (26–29). Although these inflammatory biomarkers are consistently associated with NAFLD/MASLD, their diagnostic performance is often limited, with area under the AUC values generally below 0.75, indicating moderate diagnostic accuracy.

Insulin resistance-related biomarkers have demonstrated superior diagnostic performance for MASLD, offering more reliable disease detection and progression insights. Montoro et al. demonstrated that the THR exhibited significant diagnostic value for MASLD, with an AUC of 0.747 in a cohort of 153 obese participants undergoing liver biopsy (6). Similarly, Colantoni et al. reported that THR was an effective predictor of both MASLD and its associated cardiovascular events, with AUC values of 0.721 and 0.614, respectively, in a study of 772 patients (30). The TyG index, another cost-effective surrogate marker of insulin resistance, has also been shown to be significantly associated with MASLD and its mortality (31). A recent systematic review and meta-analysis of 20 studies involving 165,725 MASLD participants found that the AUC (95% CI) of the TyG index for MASLD was 0.75 (0.71–0.79) in the summary ROC curve analysis (32). However, the TyG index has certain limitations in reflecting insulin resistance in participants with diabetes, particularly those with extreme fasting blood glucose levels. Hu et al. found that the predictive value of the TyG index for MASLD was reduced, with an AUC of 0.710 in 1,742 patients with T2DM (33). UHR has attracted increasing attention as a novel biomarker for insulin resistance (34, 35). Studies by Liu et al. and Li et al. found that UHR is significantly associated with MASLD and might serve as a novel and useful predictor for MASLD (36, 37). In our studies, UAR exhibited strong diagnostic performance for MASLD, with an AUC (95% CI) of 0.777 (0.749-0.805). These findings suggest that UAR may be a novel biomarker for MASLD. The mechanism underlying UAR’s good diagnostic ability for MASLD may be linked to the promotion of insulin resistance by UA, as well as the anti-insulin resistance properties of APOA1, indicating that elevated UAR could reflect an imbalance between pro-insulin resistance and anti-insulin resistance metabolic profiles. Additionally, UAR demonstrated superior diagnostic performance for MASLD compared to UHR (AUC difference: 0.043, 95% CI: 0.019–0.067, P < 0.001). Similarly, this study also revealed a better diagnostic value of TAR than THR (AUC: 0.780 vs. 0.742, P < 0.001), which was consistent with our previous study involving 779 participants with T2DM (13). APOA1 may serve as a more effective biomarker component for MASLD compared to HDL-c. However, further direct comparative studies are necessary to clarify the relative significance of APOA1 versus HDL-c in MASLD.

### Strengths and limitations

This study is the first to investigate the independent association between UAR and the risk of MASLD in T2DM through rigorous statistical analysis, highlighting UAR as a novel biomarker for MASLD. However, it is important to recognize several limitations of this investigation. First, the study design was cross-sectional, and the lack of longitudinal follow-up limits the ability to establish a direct relationship between UAR and MASLD. Second, steatotic liver was assessed using non-enhanced CT rather than liver biopsy, which may have led to a missed diagnosis of very mild steatosis. Thirdly, the study population was enrolled from one MMC within the Chinese population, which may limit the generalizability of the findings to other populations due to potential variations in race and ethnicity. Lastly,18 variables were included in Lasso regression to identify the most relevant covariates associated with MASLD. some confounders, like medications and exercise, may have been missed.

## Conclusion

In conclusion, this study revealed an independent association between UAR and the risk of MASLD in T2DM. Furthermore, UAR exhibited strong diagnostic performance for MASLD, comparable to TAR and superior to UHR and THR. The detection of UA and APOA1 is relatively convenient and cost-effective, suggesting that UAR could be a cost-effective biomarker to help identify high-risk groups for MASLD. Further prospective and multicenter studies are necessary to validate these findings before wide-scale clinical adoption.

## Data Availability

The raw data supporting the conclusions of this article will be made available by the authors, without undue reservation.

## Supplementary material

Supplementary Figure 1 The flowchart of the study population enrollment process. Abbreviations: UAR, serum uric acid to apolipoprotein A1 ratio.
