# Supplementary figures and images for: The serum uric acid to apolipoprotein A1 ratio is independently correlated with metabolic dysfunction-associated steatotic liver disease in type 2 diabetes mellitus: findings from a single national metabolic management center cohort
Source: Front Endocrinol (Lausanne). 2025 Jun 4;16:1619003. doi: 10.3389/fendo.2025.1619003 (PMC12173911; doi:10.3389/fendo.2025.1619003)

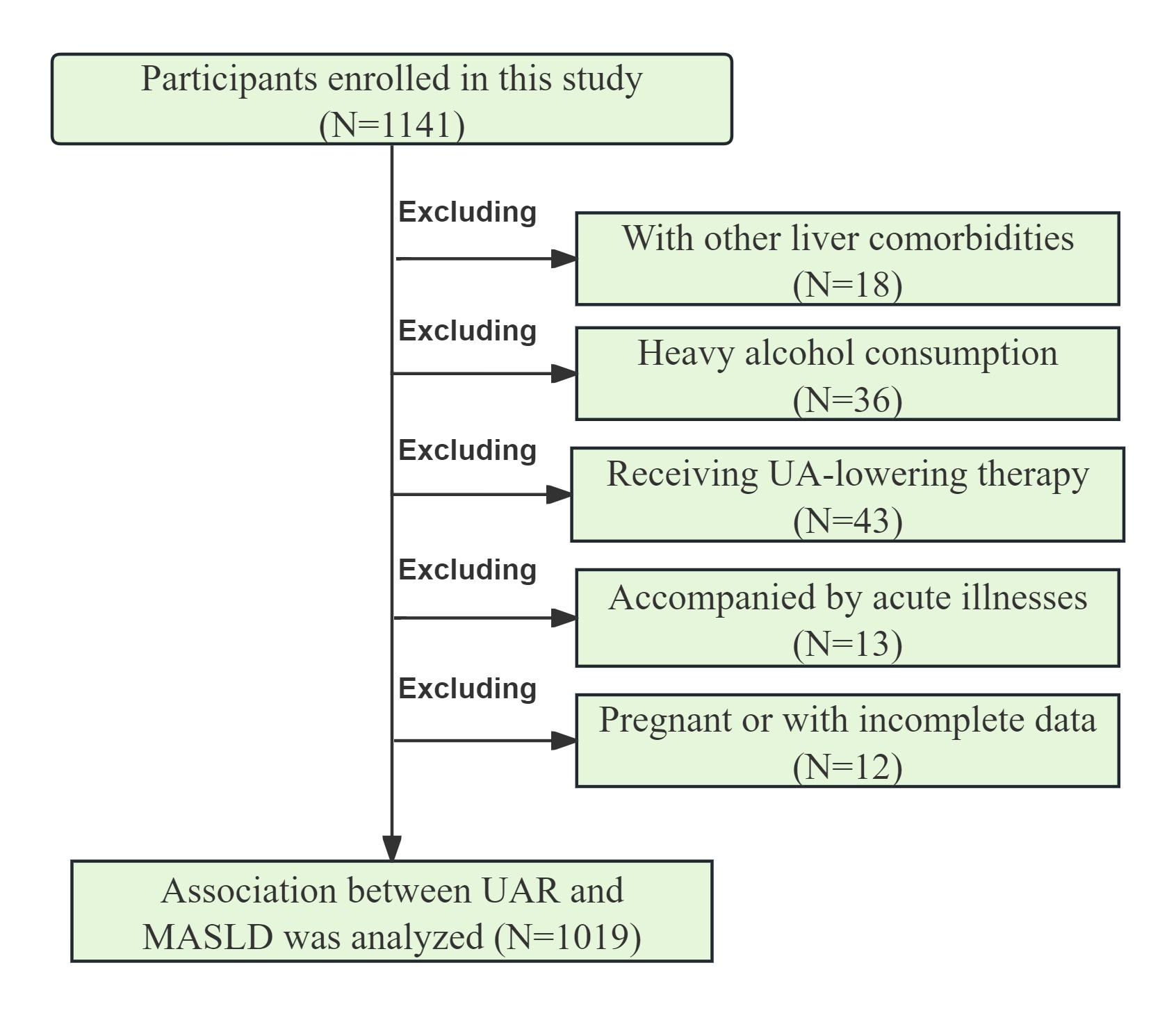

Supplement: Supplementary Figure 1 — The flowchart of the study population enrollment process. Abbreviations: UAR, serum uric acid to apolipoprotein A1 ratio. [file Image1.jpeg]
